# GlycA and CRP Are Genetically Correlated: Insight into the Genetic Architecture of Inflammageing

**DOI:** 10.3390/biom14050563

**Published:** 2024-05-08

**Authors:** Melody Kasher, Maxim B. Freidin, Frances M. K. Williams, Gregory Livshits, CHARGE Inflammation Working Group

**Affiliations:** 1 Department of Morphological Sciences, Adelson Medical School, Ariel University, Ariel 40700, Israel; melodyk@ariel.ac.il; 2 Department of Biology, School of Biological and Behavioural Sciences, Queen Mary University of London, London E1 4NS, UK; m.freydin@qmul.ac.uk; 3 Department of Twin Research and Genetic Epidemiology, School of Life Course Sciences, King’s College London, London SE1 7EH, UK; frances.williams@kcl.ac.uk; 4 Human Population Biology Research Unit, Department of Anatomy and Anthropology, Faculty of Medical and Health Sciences, Tel Aviv University, Tel Aviv 69978, Israel

**Keywords:** inflammageing, C-reactive protein, glycoprotein acetyls, cytokines, genetic association

## Abstract

Inflammageing is a condition of perpetual low-grade inflammation induced by ageing. Inflammageing may be predicted by the C-reactive protein (CRP) or by a recently described biomarker which measures N-glycosylated side chains of the carbohydrate component of several acute-phase proteins known as GlycA. The objective of this study was to examine in depth the genetic relationships between CRP and GlycA as well as between each of them and other selected cytokines, which may shed light on the mechanisms of inflammageing. Using the Olink 96 Inflammation panel, data on inflammatory mediators for 1518 twins from the TwinsUK dataset were acquired. Summary statistics for genome-wide association studies for several cytokines as well as CRP and GlycA were collected from public sources. Extensive genetic correlation analyses, colocalization and genetic enrichment analyses were carried out to detect the shared genetic architecture between GlycA and CRP. Mendelian randomization was carried out to assess potential causal relationships. GlycA predicted examined cytokines with a magnitude twice as great as that of CRP. GlycA and CRP were significantly genetically correlated (Rg = 0.4397 ± 0.0854, *p*-value = 2.60 × 10^−7^). No evidence of a causal relationship between GlycA and CRP, or between these two biomarkers and the cytokines assessed was obtained. However, the aforementioned relationships were explained well by horizontal pleiotropy. Five exonic genetic variants annotated to five genes explain the shared genetic architecture observed between GlycA and CRP: *IL6R*, *GCKR*, *MLXIPL*, *SERPINA1*, and *MAP1A.* GlycA and CRP possess a shared genetic architecture, but the relationship between them appears to be modest, which may imply the promotion of differing inflammatory pathways. GlycA appears to be a more robust predictor of cytokines compared to CRP.

## 1. Introduction

Inflammageing is defined as a state of low-grade, chronic inflammation arising from the ageing process. While it is not considered a disease, it does compromise healthy longevity, and is facilitated by immune system remodeling and cytokine alteration [1,2]. Inflammageing may contribute to the manifestation of several chronic age-related illnesses, for example, chronic kidney disease, diabetes mellitus, cancer, depression, sarcopenia, autoimmune conditions, and CVD [3,4]. Inflammageing is likely governed by complex genetic and epigenetic influences. These could stem from the dysregulation of youthful genomic networks causing innate immune cell dysfunction during ageing [5], and/or from the age-associated modification of heterochromatin and gene-specific remodeling [6]. It has been hypothesized that the genetic predisposition to a low inflammatory response and/or to heightened anti-inflammatory response in centenarians may repress the pathogenesis of inflammageing [7]. The underlying genetic architecture describing the pathogenesis of inflammageing remains unknown.

Potential indicators of inflammageing may include known biomarkers such as C-reactive protein (CRP), interleukin-6 (IL-6), and tumor necrosis factor- (TNF-α), which were correlated to ageing phenotypes; however, they were not indicative of frailty [2]. CRP, which is synthesized in the liver and released in the blood in response to inflammation [8,9], is considered to be an indicator of inflammation and is associated with chronic inflammatory diseases such as RA and CVD, among others [8,9]. While certain pro-inflammatory cytokines have been suggested to promote CRP, CRP is also suggested to regulate other cytokines [8].

Recently, glycosylated acetyls (GlycAs) have been suggested as a means to examine and identify the presence of chronic inflammation and inflammageing [8,9,10] and are able to detect aberrations in inflammatory mediators [8,11]. GlycA is the name given to the specific inflammation-related signal, which arises in the clinically measured proton nuclear magnetic resonance (NMR) spectra of serum and plasma. The GlycA NMR signal originates mainly from protons of the N-acetylmethyl group of mobile N-acetylglucosamine residues on the glycan moieties of acute phase proteins. Blood levels of these proteins usually correlate with acute-phase CRP and some other markers of systemic inflammatory responses [12].

GlycA is considered to be preferable to CRP as a biomarker because of its low intra-individual variability, its other advantages in detecting inflammation and inflammatory conditions, and it is positively associated with CRP [11]. GlycA was posited as a potential indicator of chronic inflammation and inflammageing in addition to several cytokines, including IL-6, IL-8, IL-10, IL-13, TNF-α, and IFN-γ [13,14]. Yet, the involvement of GlycA and CRP in inflammatory pathophysiology is heterogeneous, with limited similarities [15,16,17].

Since GlycA and CRP appear to regulate inflammatory mediators, or cytokines, the genetic relationship between GlycA/CRP and inflammatory mediators may reveal the genetic network involved in the pathogenesis of inflammageing. As such, the comparability of GlycA and CRP (henceforth, "biomarkers") lead us to consider the current study and clarify: 

(i) whether GlycA and CRP are genetically correlated and, if this is positive,

(ii) to define the shared genetic architecture between them that would possibly describe the extent of their comparability, and

(iii) to what extent circulating GlycA and CRP share associations with major inflammatory mediators.

## 2. Materials and Methods

### 2.1. Design of the Study

**Figure 1:**
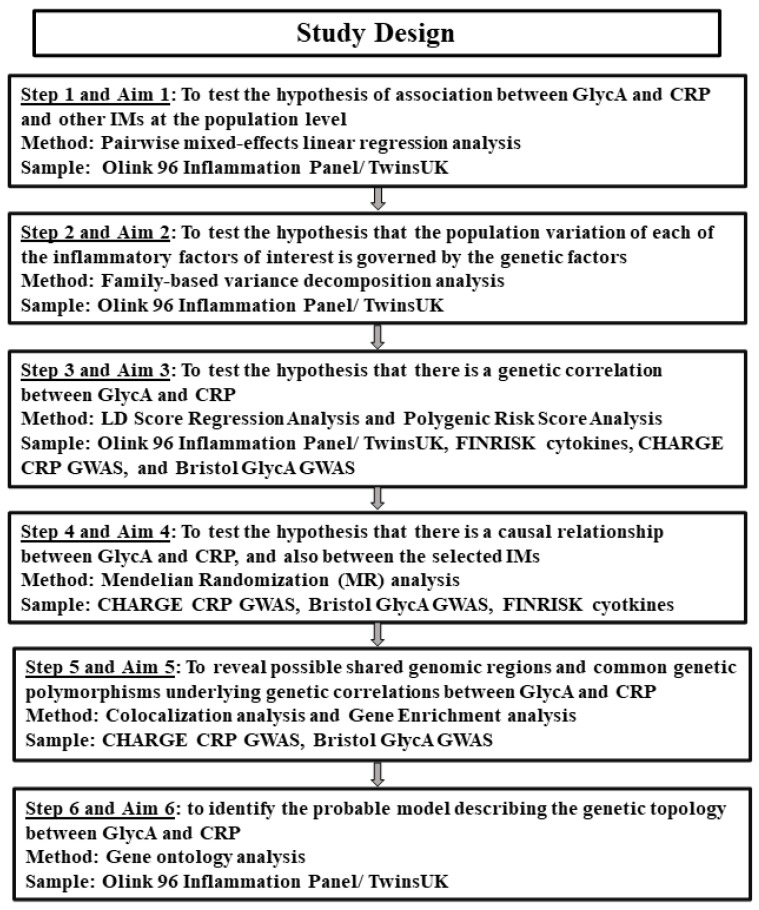
Study Design.

This study was conducted in several stages, using a few data sources, including data collected by us and publicly available sources, and implementing a number of modern genetic-epidemiological methods. The study design is diagrammatically presented in Figure 1. Following this plan, we first examined the phenotypic and putative genetic correlations between our primary phenotypes, CRP and GlycA, in a sample from TwinsUK, implementing familial-based variance component analysis. We also tested to what extent the top circulating inflammatory mediators (IMs) measured using the Olink platform [13] correlate significantly and independently with each of the primary phenotypes (biomarkers). Mixed-effects regression analysis was used to evaluate these associations.

Second, implementing a two-sample approach, we attempted to estimate the contribution of common genetic factors to examine the association between each of the two study primary biomarkers and several IMs available to this study. The polygenic risk score (PRS) assessment was the major tool to explore this question. To this aim, we also implemented the Linkage Disequilibrium Score Regression (LDSC) at this stage.

Third, we examined the genetic correlations between the CRP and GlycA established above, as well as the genetic correlations between each of them with the selected above IMs. We further considered whether these relationships exhibit a causal and/or pleiotropic nature. Mendelian randomization (MR) analysis was conducted to examine this.

At the next stage, we used colocalization methods to capture potential specific genetic factors involved in the pleiotropic relationships between the genetic correlations established and confirmed in the previous stages.

Finally, we implemented gene ontology analysis in an attempt to find the possible functional-genetic model best describing the topology between the primary phenotypes, GlycA and CRP.

The methods of analysis and data sources used at each stage of the study are indicated on the diagram and described in the following sections.

### 2.2. Datasets

The Olink96/TwinsUK dataset consisted of 1518 samples and included inflammatory mediators as well as the biomarkers, GlycA and CRP, for this cross-sectional study. The dataset was a subsample of the TwinsUK collection comprised of the longitudinal observational data of over 14,000 participants (predominantly of North European ancestry). Participants were assessed using the Olink 96 Inflammation panel [13,14]. The dataset contained 876 dizygotic and 642 monozygotic female twins with the age range of 43 and 88 years, with a mean age 64.4 ± 0.2.

Concerning the Olink 96 Inflammation panel, 96 inflammatory mediators were combined from a couple of datasets. The 96 inflammatory factors list can be found in the Olink 96 Inflammation panel [13]. Using the limit of detection (LOD) missing procedure to ascertain the extent of missingness following the merging of datasets, variables with LOD values > 30% were omitted from this study. Subsequently, 70 inflammatory mediators were available (Table S1, Supplementary Materials) which include chemokines, cytokines, among other inflammatory factors, and two primary biomarkers, GlycA and CRP. The latter two biomarkers were collected from the TwinsUK database separately. The GlycA and CRP data of the same participants found in the Olink 96 Inflammation panel were matched and formed a singular dataset that ultimately resulted in the inflammatory mediator data and biomarkers of 1518 participants.

Big data sources were accessed. The corresponding GWAS summary statistics of the inflammatory mediators were collected from the Ahola-Olli et al. study, using data from the FINRISK study and the Cardiovascular Risk in Young Finns Study, and contained 41 cytokines [15]. These cytokines were measured from 8293 Finnish participants. The respective GWAS summary statistics files consisted of over 10 million genetic variants with the covariates being age and sex [15].

The GWAS summary statistics for CRP were acquired from the Cohort for Heart and Ageing Research in Genetic Epidemiology (CHARGE) Consortium and carried out by the CHARGE Inflammation Working Group. This quantitative dataset contained 204,402 European participants gathered from 78 studies and consisted of 10 million genetic variants [16]. The GWAS summary statistics for GlycA were collected from 115,078 European participants and comprised over 12 million genetic variants, [17].

### 2.3. Statistical Analysis

Basic statistical analyses were carried out using R (Version 4.2.3; R Core Team). Mixed-effects regression analyses were conducted using the *lmer* function from the *lme4* statistical package for R. This model was used to generate a linear regression mixed-effects model considering familial genetic effects, which tends to result in a decrease in residual error. The heritability of each inflammatory mediator was estimated using the FVCA method using the MAN package [18].

### 2.4. Statistical Genetics

Genetic correlation was assessed between GlycA and CRP to examine the extent of the genetic relationship using the cross-trait Linkage Disequilibrium Score Regression (LDSC) (python implementation downloaded from https://github.com/bulik/ldsc (accessed on 1 May 2023)) [19,20]. The LD reference panel was limited to the European subset acquired from the 1000 genomes project available for download from the bulik/ldsc GitHub portal. 

### 2.5. Polygenic Risk Score Analysis

Polygenic risk score (PRS) analyses using PRSice-2 software (v2.3.5) were used to predict the potential shared genetic framework between the two biomarkers and the cytokines [21]. The PRS is computed by aggregating all trait-associated alleles in a target sample, weighted by the effect size of each allele in a base GWAS. Linkage disequilibrium (LD) is accounted for by classifying the SNPs in the LD as one to avoid the overestimation of a single marker. PRSice-2 screens for the optimal *p*-value threshold, which would explain the maximum variance of the target phenotype. The base GWAS predicts the likelihood of occurrence or the presence of the target trait. Each cytokine GWAS was used at the base GWAS and the biomarkers (GlycA/CRP) were each used as the target phenotype.

### 2.6. Mendelian Randomization (MR)

The major purpose of conducting the MR analysis was to test the hypothesis regarding a causal relationship between the exposure, phenotype X (e.g., an IM), and the primary phenotype of interest, Y—the outcome (e.g., GlycA). This analysis utilizes the principles of instrumental variable analysis assuming that the specific genetic variant *g_i_* serves as the instrument [22]. Testing the entire hypothesis relies on the three basic assumptions: (1) The relevance assumption is that, to serve as a valid instrument for the causal effect of an exposure on an outcome, the instrumental variable(s) [defined IV(s)] must be associated with the exposure phenotype only. (2) In the exclusion assumption, there is no direct association between the IV(s) and the outcome variable, the association is mediated by the exposure. (3) In the independence assumption, IV(s) is (are) independent of other factors which affect the outcome, i.e., the selected IVs must be independent from confounding factors.

In this study, MR was carried out using the *MendelianRandomization* package in R for GWAS summary statistics [23]. Two MR approaches were utilized to satisfy different assumptions, including inverse variance weighted (IVW) and the MR Egger approaches. The IVW approach is considered to be the most efficient method and is suggested as the primary method of analysis, but it is also susceptible to horizontal pleiotropy [22]. Once the association between the IV and the outcome variable is established (the relevance assumption), it is important that the exclusion-restriction assumption is satisfied. The MR Egger method examines the IV assumptions, and it was therefore subsequently implemented for its robustness in that it tests for horizontal pleiotropy by providing the MR Egger intercept while simultaneously presenting the MR Egger estimate. MR Egger additionally provides the I^2^Gx statistic, which measures the bias and validity of the instrumental variables selected [24]. Both approaches (IVW and Egger) test for the heterogeneity of the instrumental variables [22].

Instrumental variables (IVs), or genetic variants of the exposure trait, were chosen following the compilation of the GWAS summary statistics of the exposure and outcome phenotypes. A *p*-value threshold of <5.00 × 10^−8^ was applied. Subsequently, LD clumping was performed to remove the SNPs in the LD by utilizing the *ld_clump* function available in the *MRCIEU/ieugwasr* R package [25] with a parameter of clumping R^2^ = 0.01. Finally, allele harmonization was performed [22] prior to running the analysis.

The most prominent (top 8) statistically significant associations between GlycA/CRP and the cytokines were considered for MR analyses.

### 2.7. Colocalization

Colocalization analysis was implemented to find the possible *shared* causal genes/SNPs between two phenotypes. The *coloc.abf* function from the *coloc* R package by Wallace et al. uses Bayesian statistical modelling and on GWAS summary statistics [26,27]. The colocalization analysis was limited to the association between GlcyA and CRP, to determine their shared genomic regions and potentially shared SNPs. The advanced *coloc* package tests genomic regions and produces posterior probabilities (PPs) corresponding to five hypotheses between two traits of interest, which were described and presented by Wallace et al. [26]. We were particularly interested in the PP of H4 ≥ 75%, which was defined as high and strong, which estimates the PP of colocalization arising from shared common causal SNPs [26]. The tested genomic regions were selected and restricted to LD blocks [28]. 

Proposed SNPs with higher posterior probabilities generated from the SNP.PP.H4 output were considered for gene set enrichment analysis. Gene set enrichment analysis was carried out using the Functional Mapping and Annotation (FUMA) GWAS platform [29] to define the genes in common between the two phenotypes.

### 2.8. Gene Ontology

Gene ontology (GO) enrichment analysis was carried out to identify at least one model that would describe the genetic topology of one GO term or node and can be calculated under three classes: the biological process, molecular function, and cellular component [30]. Each GO term explains a cluster of genes that contribute to the process, function, or component examined. Thus, we used the *topGO R* package in *Bioconductor* [31] to identify highly associated GO terms under the three classes between GlycA and CRP. Algorithmic parameters were set to “classic” and the Fisher’s exact test statistic was assessed.

## 3. Results

### 3.1. Phenotypic Correlations

CRP was assessed as to whether it could predict GlycA, while age was considered as a covariate, and a mixed-effects multivariate model was implemented to account for the familial relationship of the twin pairs. The analysis suggested that CRP was highly and significantly associated with GlycA (β = 0.1255 ± 0.0197, *p* = 2.17 × 10^−10^), while controlling for age.

Next, age-adjusted correlations between GlycA and the panel of inflammatory mediators were computed, and were computed similarly between CRP and the inflammatory mediators (Tables S2 and S3, Supplementary Materials). Correlations were estimated 70 times with each of GlycA’s and CRP’s levels, so the Bonferroni correction was α = 0.05/70= 0.0007. We identified 27 significant correlations between GlycA and the inflammatory mediators (Table S2, Supplementary Materials), ranging between −0.1279 and +0.2368. The most significantly associated inflammatory mediators were HGF (β = 0.2368 ± 0.0260, *p*-value < 2.00 × 10^−16^), IL18R1 (β = 0.2325 ± 0.0244, *p*-value < 2.00 × 10^−16^), OSM (β = 0.2153 ± 0.0255, *p*-value < 2.00 × 10^−16^), TNFSF14 (β = 0.2049 ± 0.0251, *p*-value = 7.07 × 10^−16^), VEGFA (β = 0.1965 ± 0.0259, *p*-value = 5.32 × 10^−14^), CCL3 (β = 0.1844 ± 0.0254, *p*-value = 6.14 × 10^−13^), CCL23 (β = 0.1844 ± 0.0254, *p*-value = 6.14 × 10^−13^), and FGF21 (β = 0.1774 ± 0.0255, *p*-value = 4.78 × 10^−12^) (Table S2, Supplementary Materials). CRP demonstrated 20 significant correlations with inflammatory mediators (Table S3, Supplementary Materials), ranging between −0.1426 and +0.2553. The most significantly associated inflammatory mediators were IL6 (β = 0.2553 ± 0.0382, *p*-value = 3.81 × 10^−11^), IL18R1 (β = 0.1618 ± 0.0274, *p*-value = 4.29 × 10^−9^), VEGFA (β = 0.1587 ± 0.0289, *p*-value = 4.65 × 10^−8^), OSM (β = 0.1539 ± 0.0283, *p*-value = 6.22 × 10^−8^), CCL19 (β = 0.1462 ± 0.0274, *p*-value = 1.10 × 10^−7^), CSF1 (β= 0.1470 ± 0.0283, *p*-value = 2.39 × 10^−7^), DNER (β = −0.1426 ± 0.0282, *p*-value = 4.75 × 10^−7^), and HGF (β = 0.1477 ± 0.0297, *p*-value = 7.31 × 10^−7^) (Table S3, Supplementary Materials).

Subsequently, the eight inflammatory mediators most significantly associated with GlycA and CRP, respectively, were subjected to a mixed-effects multivariate regression model to examine their independent and combined associations, respectively, taking into account familial relationships and age (Table 1 and Table 2). The step function was used to implement a stepwise approach and identify the most optimal model by considering the Akaike information criterion. Both regression models showed high overall statistical significance (*p* < 2.2 × 10^−16^) and they explained 8% and 10% of CRP and GlycA variation, respectively. Interestingly, only a few and different cytokines were retained in these analyses, and age had a significant independent association only with GlycA.

### 3.2. Heritability Rate and Genetic Correlation

Given the highly significant association between GlycA and CRP, the extent of their genetic association was also examined. First, the heritability of each marker was evaluated by implementing familial-based variance decomposition analysis, which revealed modest significant estimates: 0.2971 ± 0.0802 (*p* = 2.11 × 10^−4^) for GlycA and 0.2786 ± 0.0246 (*p* = 1.12 × 10^−29^) for CRP. The genetic correlation between the two biomarkers was Rg = 0.4397 ± 0.0854 (*p* = 2.60 × 10^−7^).

### 3.3. Polygenic Risk Score Analysis

We further assessed the shared genetic framework using PRSice-2. PRSice-2 revealed that 23 cytokines and CRP (PRS.R^2^ = 0.0084, *p*-value = 1.46 × 10^−4^) appeared to genetically predict GlycA (Table 3), and that 17 cytokines genetically predicted CRP (Table 3). Among these were 11 cytokines commonly associated with both biomarkers (Table 3): GCSF, GROA, HGF, IL5, IL7, IL9, IL10, MIG, MIP1α, TNFα, and TNFβ. The magnitudes of the PRS.R^2^ between GlycA and these inflammatory factors were between 0.0023 for TNFβ and 0.0050 for IL5 (Table 3). The value of PRS.R^2^ between CRP and the same inflammatory factors ranged between 0.0010 for MIP1α and 0.0022 for HGF. The magnitude of the PRS results were twice as high on average, where GlycA was the target phenotype when compared to CRP.

### 3.4. Causality and Mendelian Randomization

MR was conducted to detect the causality between the biomarkers and their significantly correlated inflammatory mediators, following the Bonferroni correction. Further, we only assessed those cytokines with available GWAS summary statistics from the Finnish sample. Using the IVW approach, GlycA and CRP appeared to causally predict each cytokine examined (Tables S5 and S6, Supplementary Materials).

Upon further investigation, by implementing the MR Egger approach to distinguish between causal and pleiotropic effects, a causal effect was only seen between GlycA and VEGF (β = 0.291, 95%CI= 0.016 to 0.567, *p*-value = 0.038), with no evidence of horizontal pleiotropy (Table 4). The genetic relationship between GlycA and most of the selected cytokines, including HGF (*p*-value = 0.002), IL6 (*p*-value = 0.008), IL7 (*p*-value = 0.002), TNFα (*p*-value = 0.043), as well as that with CRP (*p*-value = 0.003), appeared to be explained by horizontal pleiotropy (Table 4). No causal relationship was evident between CRP and the associated cytokines with the genetic associations explained by horizontal pleiotropy with HGF (*p*-value = 0.001), IL6 (*p*-value = 0.035), and VEGF (*p*-value = 0.037) (Table 5). 

### 3.5. Colocalization Analysis and Gene Enrichment

Colocalization and gene enrichment analysis identified shared pleiotropic SNPs and their corresponding genes between GlycA and CRP. While the significant SNPs found in the GWAS profile of each biomarker had some key differences, some shared genes were apparent (Table 6). The reported results were restricted to evidence of high posterior probabilities (≥75%) for shared SNPs between the biomarkers (H4), or distinct causal SNPs on the same gene (H3). The colocalization analysis revealed 17 genomic regions in the colocalization between GlycA and CRP found on chromosomes 1, 2, 6, 7, 8, 9, 11, 14, 15, and 19 (Table 6). While intronic and intergenic SNPs were seen, interestingly, five exonic SNPs were also evident and shared between the two biomarkers (Table 6). On chromosome 1, between base pairs 151,538,881 and 154,770,403, a nonsynonymous exonic SNP was evident with a PP.H4 of 98.1% and corresponded to the gene, *IL6R* (Table 6). Subsequently, on chromosome 2 between base pairs 110,572,432 and 113,921,856, another nonsynonymous exonic SNP was observed with a PP.H4 of 99.9% and was harbored by the *GCKR* gene (Table 6). Next, another shared exonic SNP was found on chromosome 7 ranging between base pairs 71,874,885 and 73,334,602 with a PP.H3 of 100%, and was noted near the gene *MLXIPL* (Table 6). The final two exonic SNPs were located on chromosomes 14 and 15, and spanned the base pair regions 94,325,285–95,750,867 and 42,776,399–44,198,049, respectively, and were harbored by the genes *SERPINA1* and *MAP1A*, respectively (Table 6). The posterior probabilities of H4 were 99.8% and 85.0%, respectively (Table 6).

### 3.6. Comparative Enrichment—Gene Ontology

Subsequently, comparative gene ontology was performed to assess the potential similarity using gene ontology terms between GlycA and CRP. The GO terms were mapped and annotated by each variable and calculated using Fisher’s exact test to determine the most likely represented GO term associated with each variable by class. 

The Fisher test revealed that GlycA and CRP significantly shared GO terms in each class, biological process, molecular function, and cellular component (Table S7, Supplementary Materials). Moreover, each GO term was seen in the first node, except for molecular function in CRP, where the same GO term was reflected in the second node (Table S7, Supplementary Materials). In examining the biological process class, GlycA and CRP were significantly associated with GO:0007156, which denotes homophilic cell adhesion via a plasma membrane (*p*-values = 3.60 × 10^−6^ and 1.40 × 10^−5^, respectively) (Table S7, Supplementary Materials). Next, the molecular function class showed that the GO:0005515 term was significantly associated with GlycA and CRP (*p*-values = 4.00 × 10^−8^ and 5.90 × 10^−7^, respectively), and demonstrated protein binding (Table S7, Supplementary Materials). Lastly, the cellular component class indicated that GlycA and CRP were significantly associated with the GO:0005829 term (*p* = 4.00 × 10^−8^ and 5.90 × 10^−7^, respectively), which expressed the cytosol (Table S7, Supplementary Materials).

## 4. Discussion

### 4.1. Overview

This study has defined the relationship between the biomarkers of inflammageing, GlycA and CRP, and other inflammatory mediators. GlycA and CRP were significantly correlated both phenotypically and genetically; however, differences were also apparent. Genetically, they posed a significant genetic correlation indicating some shared genetic architecture which was confirmed by the PRS estimates and colocalization analysis. Their genetic relationship was found to be horizontally pleiotropic following the Mendelian randomization analysis, with some key genes shared; however, genetic variation was still evident in their respective genetic profiles. Associations of cytokines with GlycA tended to be higher compared to those with CRP, and the lists of the associated cytokines partially overlapped. These results were in agreement with the results of the Mendelian randomization analyses. GlycA was consistently superior to CRP in its association with inflammatory mediators when examining the magnitude of association and overall collection of statistically significant associations. 

### 4.2. Cytokines of Interest

Interestingly, in the case of GlycA, the results suggested possible causality for IL-10 and -12, while the others factors shared common pleiotropic effects. Of these, the most prominent were pleiotropic correlations with IL-6, IL-7, and HGF. CRP, on the other hand, displayed only pleiotropic relations with other inflammatory mediators, in particular, the most significant was that with IL-6 and HGF. Notably, IL-7 contributes to the elicitation of IL-17 [32]. IL-17 is involved in T cell activation and is involved in the pathogenesis of inflammatory conditions, such as rheumatoid arthritis and psoriasis [33]. HGF is involved in various inflammatory pathways and inflammatory conditions, while exhibiting anti-inflammatory properties [34]. IL-6 is a well-established key cytokine in autoimmune conditions, chronic inflammation, and infections [35] and is also a target for immunomodulatory treatment.

One of the highly significant (*p* = 2.46 × 10^−8^, Table 2) correlations to CRP was its correlation with DNER (Delta- and Notch-like Epidermal Growth Factor Receptor). This correlation is not entirely clear since DNER is predominantly expressed in the nervous system and in various tumors, and its soluble form in the serum is a marker for a number of tumors but not for inflammation [36]. However, as we tested the whole battery of OLINK markers without a priori assumption, we could not neglect this result, which survived the multiple testing correction, and in terms of statistical significance, was the top marker. It displayed an association even more significant than that between CRP and IL6 (*p* = 8.24 × 10^−6^). We do not have a clear explanation for this correlation. However, we may note that although the pro- or anti-inflammatory functions of Notch signaling were not shown, it has been shown to modulate inflammatory conditions such as sepsis, and therefore may significantly impact the course of disease [37]. This, however, does not concern DNER. It has been reported that DNER is the actual target for anti-Tr antibodies [38], which in turn could be associated with paraneoplastic cerebellar degeneration and Hodgkin disease [39]. To further understand the metabolic bridge (if there is any) between the anti-Tr antibodies and CRP would suggestively require clarification and further study.

### 4.3. GlycA vs. CRP

A highly significant (*p* = 2.17 × 10^−10^) association was seen between the circulating levels of GlycA and CRP. This is in agreement with one previously published study, wherein the modest sample (58 participants) of individuals who were obese manifested a significant correlation between their plasma levels (r = 0.46; *p* < 1 × 10^−3^) [40]. The multiple regression analyses suggest differing mechanisms for GlycA and CRP, whereby they were significantly associated with different inflammatory mediators, while demonstrating similar regression coefficients. However, despite their similarities, GlycA and CRP appeared to employ differing inflammatory pathways, which yield to GlycA’s consistency, unlike CRP [40]. While CRP’s response is more apparent in the early stages of the disease, GlycA’s response may be more indicative in the acute phases of the disease [9]. Unlike CRP, GlycA posits low intra-individual variability [9].

CRP is a classical marker of inflammation and infection. GlycA has been relatively recently suggested as a superior predictor of chronic inflammatory illness as well as autoimmune disorders [9]. It may provide a more accurate predictor of CVD risk, RA, type 2 diabetes, and other chronic and/or autoimmune conditions [9]. Moreover, when GlycA was compared to CRP in depicting the metabolomic profile dictating CVD risk, GlycA appeared as the more sensitive and accurate determinant [41]. Subsequently, GlycA, unlike CRP, is linked with the gut microbiome, which would otherwise predict disease among other conditions [41]. GlycA and hsCRP showed similarities along short-term measurements and associations with inflammatory mediators [17]. However, GlycA was more indicative of chronic inflammation, which is maintained by its long-term, perpetual, and consistent presence, unlike CRP, which reflected a short-term response to acute inflammation [17]. Similar to our findings, a report demonstrated that GlycA was statistically associated with some cytokines, and suggested that GlycA may encapsulate and possibly represent cytokine alteration [42].

Elevated GlycA has been observed in low-grade chronic inflammation, and an increase is GlycA levels is indicative of hospitalization and increased mortality arising from infection [42]. The characteristics apparent in GlycA essentially depict the description of inflammageing [8]. Otherwise, the current suggested indicators of inflammageing include CRP, IL6, IL8, and TNF [43]. 

### 4.4. Genes of Interest

While CRP and GlycA were indicative of horizontally pleiotropic relationships with other cytokines, they also exhibited a horizontally pleiotropic relationship with each other. Through conducting a colocalization analysis, several shared genomic regions emerged. Interestingly, five genomic regions yielded shared nonsynonymous exonic SNPs. These five mutations were annotated to the following five genes: *IL6R*, *GCKR*, *MLXIPL*, *SERPINA1* and *MAP1A*.

The functional role of IL6R is apparent in cancer, cell differentiation, and inflammation [44]. Importantly, both *GCKR* and *IL6R* were reported in metabolic syndrome. In particular, the rs1260326 polymorphism in *GCKR* showed a 21% increase in susceptibly to metabolic syndrome [45,46]. Metabolic syndrome may be considered to be a culmination of cardiometabolic abnormalities with a genetic foundation that leads to several disorders including, but not limited to, cardiovascular complications, diabetes, neurological complications, and an overall proinflammatory state. Such findings substantiate CRP, and recently GlycA, as biomarkers that may be used to predict metabolic syndrome [47,48]. 

The particular polymorphism in *IL6R*, rs2228145, contributes to the genetic predisposition of nonalcoholic steatohepatitis, and a variation of hematological levels [49,50]. Interestingly, rs2228145 was also associated with chronic autoimmune conditions including asthma, coronary heart disease, rheumatoid arthritis, and type 1 diabetes [51,52,53,54]. On a molecular level, the acute phase of CRP is related to the production of soluble IL6R by threefold, which may subsequently contribute to the inflammatory response [55]. The relationship between IL6R and GlycA is still poorly understood. The rs1260326 polymorphism mapped to *GCKR* was reportedly associated with non-alcoholic fatty liver disease and cardiovascular disease-related phenotypes [56,57]. This polymorphism is of interest because it was significantly associated not only with CRP but also with GlycA [58,59]. 

The colocalization analysis identified other interesting candidate genes associated with exonic SNPs, which may have an important clinical orientation. The first is *MLXIPL*, a gene that is deleted in Williams-Beuren syndrome, a multisystem developmental disorder, and is associated with non-alcoholic fatty liver disease [60] as well as metabolic syndrome [61]. Essentially, *MLXIPL* is responsible for the modulation of hepatic carcinoma [62]. The *MLXIPL* gene’s function is related to the carbohydrate response element-binding protein, and in particular, to the rs3812316 polymorphism, to blood triglyceride levels, cardiovascular disease, and metabolic alterations [63,64]. Interestingly, the molecular function in the comparative gene ontology analysis in our study detected protein binding as a shared characteristic between GlycA and CRP. *MLXIPL* was also suggestively associated with CRP [61]. However, to the best of our knowledge, the association between *MLXIPL* and GlycA has not yet been reported in the literature.

Another notable gene was *SERPINA1. SERPINA* regulates immune function and inhibits proteases [65]. The rs28929474 polymorphism near *SERPINA1* increases susceptibly to childhood asthma and was previously noted to contribute to the shared genetic relationship between rheumatoid arthritis and osteoporosis [66,67]. Alpha 1 Antitrypsin deficiency, which is expressed by the *SERPINA1* gene, is associated with elevated levels of CRP, thus suggesting an inflammatory component [68]. However, this study reported the strong association of GlycA with *SERPINA1* variants, for the first time.

Another gene of interest, *MAP1A*, has not been previously associated with both CRP and GlycA. *MAP1A* is functionally involved in the development of neuronal components, such as axons and dendrites [69].

### 4.5. Limitations

The TwinsUK sample may be too small for full genetic analyses. Thus, genetic findings involving the cytokines were underpowered, and further larger studies may reveal further contrasts. Moreover, the number of inflammatory mediators available from the Olink panel was limited to 70 due to insufficient data, as was tested by the LOD missing analysis. Only a limited number of GWASs were available in the Finnish sample. Despite this difference, some GWASs available from the Finnish sample were generalized to fit several mediators and the most prominent cytokines were nevertheless available. It should also be mentioned that despite the fact that >70 inflammatory mediators were examined in this study, some others, such as *interleukin-1\u03b2 (IL-1\u03b2)* or *interferon-\u03b3 (IFN-\u03b3)*, could also be of interest, but were not included in this project.

## 5. Conclusions

Both GlycA and CRP were significantly associated with mutations that increase susceptibility to metabolic syndrome and were also associated with inflammatory diseases and chronic conditions which were ascribed to inflammageing. Still, GlycA appears to be more explicit in describing the cytokines, as its associations were more comprehensive in magnitude and proportion when compared to CRP. Of interest was the overall shared architecture between GlycA and CRP, which might explain their consistencies and potentially isolate genes that may be attributed to inflammageing. Despite their shared genetic architecture, and that there is variation is cytokine prediction, we therefore speculate that GlycA and CRP potentially emulate different inflammatory pathways.

## 6. Possible Clinical Implications

Our above conclusion, based on the extensive genetic analysis, is in good agreement with Tebar et al.’s study [70] which evaluated the cross-sectional association of CRP and GlycA with carotid artery plaque (CAP), obesity, and some other conditions from the ELSA-Brasil adult cohort. The analysis included 4126 participants with a median age of 50 years old. The authors concluded that their findings suggest potentially different biological pathways between GlycA and CRP, despite the correlation between them. They believe that GlycA is associated mostly with inflammation and carotid atherosclerosis, whereas high CRP was more associated with obesity. Clearly more studies are needed to confirm these conclusions, but if they are positive, this means that elevated levels of GlycA and CRP could be used for differential diagnostic and prognostic implications in clinical practice.

## Figures and Tables

**Table 1:** Mixed multiple regression analysis where the GlycA level is the dependent variabe and inflammatory mediators are predictors.

Independent Variables	Estimate	SE	*p*-Value
**Intercept**	0.0107	0.0297	7.19 × 10^−1^
**IL18R1**	0.1724	0.0251	9.56 × 10^−12^
**OSM**	0.1353	0.0250	7.35 × 10^−8^
**FGF21**	0.1009	0.0251	6.02 × 10^−5^
**Age**	0.0947	0.0302	1.81 × 10^−3^

Multiple R-squared: 0.1028, *p*-value: <2.2 × 10^−16^; all variables were standardized.

**Table 2:** Mixed multiple regression analysis where the CRP level is the dependent variable and inflammatory mediators are predictors. CRP Results: Multiple R-squared: 0.0759, *p*-value: <2.2 × 10^−16^; all variables were standardized prior to analysis.

Independent Variables	Estimate	SE	*p*-Value
**Intercept**	0.0763	0.0400	5.64 × 10^−2^
**IL18R1**	0.1185	0.0436	6.68 × 10^−3^
**IL6**	0.1805	0.0403	8.24 × 10^−6^
**VEGFA**	0.1036	0.0464	2.58 × 10^−2^
**DNER**	−0.2223	0.0396	2.46 × 10^−8^

**Table 3:** Polygenic risk score (PRS) results of the analysis of GlycA and CRP. Only significant PRS results are shown here (*p* < 0.05). Full PRS results including all 41 inflammatory mediators can be found in Table S4, Supplementary Materials.

Base GWAS	Target	Threshold	PRS.R^2^	Full.R^2^	Null.R^2^	Coefficient	Standard Error	*p*-Value	Number of SNP
**B_NGF**	GlycA	0.3904	0.0047	0.0167	0.0120	−197.16	69.70	4.73 × 10^−3^	86,767
**CRP**	GlycA	0.0525	0.0084	0.0205	0.0120	479.34	125.94	1.46 × 10^−4^	24,677
**GCSF**	GlycA	0.2646	0.0029	0.0150	0.0120	−191.20	85.64	2.57 × 10^−2^	69,225
**GROA**	GlycA	0.0026	0.0045	0.0166	0.0120	16.04	5.76	5.43 × 10^−3^	1632
**HGF**	GlycA	0.0047	0.0032	0.0152	0.0120	29.60	12.68	1.97 × 10^−2^	3025
**IL10**	GlycA	0.3433	0.0030	0.0151	0.0120	−212.77	93.28	2.27 × 10^−2^	81,302
**IL12**	GlycA	0.1460	0.0049	0.0170	0.0120	−184.79	63.64	3.74 × 10^−3^	46,700
**IL16**	GlycA	0.0003	0.0032	0.0153	0.0120	3.93	1.67	1.86 × 10^−2^	186
**IL17**	GlycA	0.0003	0.0032	0.0152	0.0120	−7.56	3.23	1.95 × 10^−2^	277
**IL18**	GlycA	0.0000	0.0023	0.0144	0.0120	0.23	0.11	4.64 × 10^−2^	6
**IL1B**	GlycA	0.0069	0.0025	0.0145	0.0120	−19.80	9.65	4.03 × 10^−2^	3979
**IL4**	GlycA	0.0174	0.0027	0.0147	0.0120	−49.91	23.22	3.17 × 10^−2^	8899
**IL5**	GlycA	0.0002	0.0050	0.0171	0.0120	4.56	1.55	3.38 × 10^−3^	144
**IL7**	GlycA	0.0679	0.0029	0.0150	0.0120	−65.97	29.43	2.51 × 10^−2^	25,883
**IL9**	GlycA	0.0504	0.0023	0.0143	0.0120	−53.39	27.05	4.85 × 10^−2^	20,691
**MCP3**	GlycA	0.0035	0.0052	0.0173	0.0120	−11.94	3.98	2.74 × 10^−3^	2199
**MCSF**	GlycA	0.0001	0.0038	0.0158	0.0120	−1.93	0.76	1.08 × 10^−2^	59
**MIG**	GlycA	0.0487	0.0048	0.0168	0.0120	−72.30	25.28	4.29 × 10^−3^	20,112
**MIP1α**	GlycA	0.0003	0.0043	0.0164	0.0120	5.47	2.00	6.44 × 10^−3^	248
**PDGF**	GlycA	0.0449	0.0029	0.0150	0.0120	86.18	38.51	2.53 × 10^−2^	19,340
**SCGF**	GlycA	0.0004	0.0042	0.0162	0.0120	6.80	2.54	7.43 × 10^−3^	350
**TNFα**	GlycA	0.0032	0.0042	0.0162	0.0120	−17.93	6.68	7.36 × 10^−3^	2036
**TNFβ**	GlycA	0.0051	0.0023	0.0143	0.0120	−11.39	5.78	4.88 × 10^−2^	2834
**VEGF**	GlycA	0.0049	0.0025	0.0146	0.0120	−23.60	11.32	3.71 × 10^−2^	3167
**CTACK**	hsCRP	0.0122	0.0013	0.0021	0.0008	737.42	315.68	1.95 × 10^−2^	6432
**GCSF**	hsCRP	0.0002	0.0013	0.0022	0.0008	151.87	63.17	1.62 × 10^−2^	182
**GROA**	hsCRP	0.0001	0.0017	0.0026	0.0008	32.43	11.76	5.83 × 10^−3^	42
**HGF**	hsCRP	0.0388	0.0022	0.0030	0.0008	2526.22	819.79	2.07 × 10^−3^	16,920
**IL10**	hsCRP	0.0063	0.0014	0.0022	0.0008	816.14	331.20	1.38 × 10^−2^	3851
**IL13**	hsCRP	0.0098	0.0018	0.0026	0.0008	793.16	286.06	5.58 × 10^−3^	5469
**IL1RA**	hsCRP	0.0020	0.0021	0.0029	0.0008	390.15	128.89	2.48 × 10^−3^	1435
**IL5**	hsCRP	0.0000	0.0011	0.0019	0.0008	4.33	2.01	3.12 × 10^−2^	1
**IL6**	hsCRP	0.0003	0.0012	0.0020	0.0008	−151.93	67.34	2.41 × 10^−2^	209
**IL7**	hsCRP	0.0023	0.0013	0.0021	0.0008	311.91	132.34	1.85 × 10^−2^	1592
**IL9**	hsCRP	0.0192	0.0009	0.0017	0.0008	773.62	392.08	4.85 × 10^−2^	9498
**MCP1**	hsCRP	0.0954	0.0011	0.0019	0.0008	−2693.09	1229.10	2.85 × 10^−2^	33,867
**MIG**	hsCRP	0.0000	0.0011	0.0020	0.0008	6.92	3.13	2.71 × 10^−2^	2
**MIP1α**	hsCRP	0.0154	0.0010	0.0019	0.0008	727.29	345.39	3.53 × 10^−2^	7966
**TNFα**	hsCRP	0.0045	0.0013	0.0022	0.0008	−449.89	186.96	1.62 × 10^−2^	2691
**TNFβ**	hsCRP	0.0019	0.0010	0.0018	0.0008	−162.52	79.27	4.04 × 10^−2^	1146
**TRAIL**	hsCRP	0.0011	0.0018	0.0026	0.0008	−391.97	141.91	5.77 × 10^−3^	857

**Table 4:** The Mendelian randomization with GlycA as the exposure variable. The MR Egger method suggests that the majority of the inflammatory mediators are in horizontal pleiotropy with GlycA.

Outcome	Instrumental Variables	Estimate	SE	95% Confidence Interval	*p*-Value	MR Egger Intercept *p*-Value	I^2^Gx	Heterogeneity
**hsCRP**	21	0.001	0.036	−0.068, 0.071	0.975	0.003	97.7%	0.5688
**FGF**	38	0.114	0.107	−0.096, 0.324	0.289	0.062	98.1%	0.8006
**HGF**	41	0.113	0.105	−0.093, 0.319	0.284	0.002	98.0%	0.9371
**IL6**	41	0.070	0.096	−0.118, 0.257	0.466	0.008	98.1%	0.9201
**IL7**	36	0.030	0.147	−0.257, 0.318	0.836	0.002	97.2%	0.9599
**TNFα**	37	0.082	0.140	−0.192, 0.356	0.558	0.043	97.6%	0.9920
**VEGF**	45	0.291	0.140	0.016, 0.567	0.038	0.471	95.4%	0.9781

**Table 5:** The Mendelian randomization with hsCRP as the exposure variable. The MR Egger method suggests that the majority of the inflammatory mediators are in horizontal pleiotropy with hsCRP.

Outcome	Instrumental Variables	Estimate	SE	95% Confidence Interval	*p*-Value	MR Egger Intercept *p*-Value	I^2^Gx	Heterogeneity
**HGF**	34	0.146	0.424	−0.685, 0.977	0.731	0.001	98.7%	1.0000
**IL6**	46	0.120	0.091	−0.057, 0.298	0.184	0.035	97.3%	0.9170
**IL10**	38	0.215	0.139	−0.057, 0.487	0.122	0.220	93.2%	0.9979
**TNFα**	35	0.360	0.223	−0.077, 0.796	0.106	0.300	80.5%	1.0000
**VEGF**	35	0.077	0.115	−0.149, 0.304	0.502	0.037	96.5%	0.9868

**Table 6:** The GlycA and CRP genetic colocalization analysis results. Seventeen genomic regions showed the colocalization of the SNP with *p* < 5 × 10^−8^ for both hsCRP and GlycA. These regions specifically indicate shared SNPs, or at least SNPs in the same genomic regions.

Cytokine in Colocalization with GlycA	Genomic Region Chromosome: Base Pairs	Gene (SNP) Function	GlycA *p*-Value	hsCRP *p*-Value	PP.H4 (Posterior Probability of Shared Causal SNP) or PP.H3 (of SNPs in Same Region)
**hsCRP**	Chr1: 25516845–27401867	*ZDHHC18* rs75460349 intronic	5.20 × 10^−8^	4.88 × 10^−10^	H4: 99.0%
**hsCRP**	Chr1: 65041704–66939401	*LEPR/RN7SL854P* rs13375019 intergenic	4.40 × 10^−13^	1.35 × 10^−134^	H4: 79.3%
**hsCRP**	Chr1: 151538881–154770403	*IL6R* rs2228145 Nonsynonymous SNV, exon9	3.30 × 10^−7^	1.21 × 10^−101^	H4: 98.1%
**hsCRP**	Chr1: 247344518–249239466	*NLRP3* rs56188865 intronic	3.10 × 10^−11^	1.95 × 10^−22^	H4: 98.8%
**hsCRP**	Chr2: 110572432–113921856	*GCKR* rs1260326 Nonsynonymous SNV, exon15	2.60 × 10^−125^	5.44 × 10^−61^	H4: 99.9%
**hsCRP**	Chr2: 26894985–28598777	*IL1F10/RNU6-1180P* rs6734238 intergenic	4.00 × 10^−9^	7.46 × 10^−29^	H4: 100%
**hsCRP**	Chr6: 31571218–32682664	*HLA-DRA/HLA-DRB9* rs9268790 intergenic	1.10 × 10^−23^	8.9 × 10^−9^	H3: 96.4%
**hsCRP**	Chr7: 71874885–73334602	*MLXIPL* rs3812316 Nonsynonymous SNV, exon6	3.90 × 10^−59^	3.52 × 10^−12^	H3: 100%
**hsCRP**	Chr8: 10463197–11278998	*LINC00529* rs10481445 ncRNA_intronic	8.10 × 10^−10^	6.29 × 10^−11^	H3: 97.7%
**hsCRP**	Chr8: 11278998–13491775	*FDFT1* rs2409836 intronic	8.10 × 10^−10^	1.09 × 10^−12^	H3: 90.9%
**hsCRP**	Chr8: 7153079–9154694	*CTA-398F10.2* rs2921057 ncRNA_exonic	2.10 × 10^−10^	5.82 × 10^−10^	H3: 98.8%
**hsCRP**	Chr8: 9154694–9640787	*RP11-115J16.* rs4841133 ncRNA_exonic	2.10 × 10^−22^	3.31 × 10^−19^	H3: 100%
**hsCRP**	Chr9: 135298842–137041122	*ABO* rs543040 ncRNA_intronic	8.90 × 10^−11^	1.97 × 10^−9^	H4: 97.1%
**hsCRP**	Chr11: 12564229–13373124	*ARNTL* rs7947951 intronic	5.10 × 10^−6^	5.81 × 10^−9^	H4: 96.8%
**hsCRP**	Chr14: 94325285–95750867	*SERPINA1* rs28929474 Nonsynonymous SNV, exon7	3.80 × 10^−80^	5.47 × 10^−10^	H4: 99.8%
**hsCRP**	Chr15: 42776399–44198049	*MAP1A* rs55707100 Nonsynonymous SNV, exon4	1.50 × 10^−7^	1.53 × 10^−4^	H4: 85.0%
**hsCRP**	Chr19: 34262952–36469295	*HPN-AS1* rs2445818 ncRNA_intronic	2.60 × 10^−8^	5.71 × 10^−5^	H4: 86.7%

## Data Availability

Data are contained within the article and Supplementary Materials.

## Supplementary Materials

The following supporting information can be downloaded at: https://www.mdpi.com/article/10.3390/biom14050563/s1, Table S1: List of 70 inflammatory mediators presented from the Olink 96 panel; Table S2: Correlations between GlycA and Inflammatory Mediators; Table S3: Correlations between hsCRP and inflammatory mediators; Table S4: PRS results for GlycA and CRP; Table S5: MR GlycA as exposure using the IVW method; Table S6: MR hsCRP as the exposure, using the IVW method; Table S7: Gene Ontology results: enrichment analysis r for GlycA and CRP variables for gene ontology (GO) terms.
