# Supplementary material for: GlycA and CRP Are Genetically Correlated: Insight into the Genetic Architecture of Inflammageing
Source: Biomolecules. 2024 May 8;14(5):563. doi: 10.3390/biom14050563 (PMC11117775; doi:10.3390/biom14050563)
Supplement: Supplementary file 1 [file biomolecules-14-00563-s001.zip › biomolecules-2977830-supplementary.pdf]

GlycA and CRP are genetically correlated: insight into the genetic architecture of inflammageing.

Melody Kasher, Maxim B. Freidin, Frances MK Williams, CHARGE Inflammation Working Group, Gregory Livshits

Supplementary Material.

Table S1. List of 70 inflammatory mediators presented from the Olink 96 panel

Inflammatory Factors
TNF-related_apoptosis-inducing_ligand
Adenosine_deaminase
Axin-1
Caspase_8
Chemokine_C-C_motif_ligand_11
Chemokine_C-C_motif_ligand_19
Chemokine_C-C_motif_ligand_20
Chemokine_C-C_motif_ligand_23
Chemokine_C-C_motif_ligand_25
Chemokine_C-C_motif_ligand_28
Chemokine_C-C_motif_ligand_3
Chemokine_C-C_motif_ligand_4
chemokine_C-X3-C_motif_ligand_1
cluster_of_differentiation_244
cluster_of_differentiation_5
cluster_of_differentiation_6
cluster_of_differentiation_6
cluster_of_differentiation_8_alpha
Colony_Stimulating_Factor_1
CUB_domain_containing_protein_1
C-X-C_motif_chemokine_11
C-X-C_motif_chemokine_ligand_1
C-X-C_motif_chemokine_ligand_10
C-X-C_motif_chemokine_ligand_5
C-X-C_motif_chemokine_ligand_6
C-X-C_motif_chemokine_ligand_9
Cystatin_D
Delta_and_Notch-like_epidermal_growth-factor-related_receptor
EN-RAGE
Fibroblast_Growth_Factor-21
Fibroblast_Growth-Factor-19
Flt-3_Ligand
Hepatocyte_Growth_Factor

Inteleukin_6
Interferon_gamma
Interleukin_10
Interleukin_10RB
Interleukin_12B
Interleukin_15RA
Interleukin_17C
Interleukin_18
Interleukin_18R1
Interleukin_7
Interleukin_8
latency-associated_peptide_TGF_beta1
Leukemia_inhibitory_factor_receptor
matrix_metalloproteinases-1
matrix_metalloproteinases-10
Monocyte_Chemoattractant_Protein-1
Monocyte_Chemoattractant_Protein-2
Monocyte_Chemoattractant_Protein-4
Neurotrophin-3
Oncostatin_M
Osteoprotegerin
Programmed_death-ligand_1
Signaling_Lymphocytic_Activation_Molecule_Family_Member_1
Sirtuin_2
STAM_Binding_Protein
Stem_cell_factor
Sulfotransferase_1A1
TNF_Receptor_Superfamily_Member_9
TNF_Superfamily_Member_14
TNF-related_activation-induced_cytokine
TNF-related_weak_inducer_of_apoptosis
Transforming_growth_factor_alpha
Tumor_necrosis_factor
Tumor_Necrosis_Factor_B
Urokinase-type_plasminogen-activator
Vascular_Endothelial_Growth_Factor_A
X4E_BP1

Table S2. Correlations between **GlycA** and Inflammatory Mediators

Variable	GlycA Correlations	GlycA SE	GlycA p-values
**ADA**	0.0355	0.0259	1.70E-01
**AXIN1**	0.0368	0.0257	1.52E-01
**CASP.8**	0.0884	0.0256	5.52E-04
**CCL11**	-0.0062	0.0270	8.19E-01
**CCL19**	0.1425	0.0284	1.14E-08
**CCL20**	0.0844	0.0258	1.10E-03
**CCL23**	0.1844	0.0254	6.14E-13
**CCL25**	-0.0748	0.0272	6.11E-03
**CCL28**	-0.0709	0.0266	7.68E-04
**CCL3**	0.1844	0.0254	6.14E-13
**CCL4**	0.0908	0.0255	3.83E-04
**CD244**	0.0272	0.0257	2.91E-01
**CD40**	0.1153	0.0256	7.08E-06
**CD5**	0.1101	0.0256	1.85E-05
**CD6**	0.0210	0.0263	4.25E-01
**CD8A**	0.0191	0.0249	4.45E-01
**CDCP1**	0.0931	0.0299	1.91E-03
**CSF.1**	0.1348	0.0256	1.56E-07
**CST5**	-0.0239	0.0265	3.68E-01
**CX3CL1**	-0.0458	0.0255	7.23E-02
**CXCL1**	0.0563	0.0257	2.85E-02
**CXCL10**	0.0283	0.0259	2.76E-01
**CXCL11**	0.0938	0.0251	1.89E-04
**CXCL5**	0.0813	0.0255	1.46E-03
**CXCL6**	0.0453	0.0258	7.95E-02
**CXCL9**	0.0020	0.0282	9.42E-01
**DNER**	-0.1279	0.0256	6.81E-07
**EN.RAGE**	0.1056	0.0260	5.07E-05
**FGF.19**	0.0109	0.0259	6.74E-01
**FGF.21**	0.1774	0.0255	4.78E-12
**Flt3L**	-0.0235	0.0267	3.79E-01
**HGF**	0.2368	0.0260	<2.00E-16
**IFN.gamma**	0.0306	0.0254	2.35E-01
**IL.10RB**	0.1117	0.0255	1.28E-05
**IL.12B**	0.0843	0.0257	1.07E-03

**IL.15RA**	0.1108	0.0281	8.54E-05
**IL.17C**	-0.0201	0.0276	4.68E-01
**IL.18R1**	0.2325	0.0244	<2.00E-16
**IL10**	0.0693	0.0261	7.96E-03
**IL18**	0.0850	0.0256	9.18E-04
**IL6**	0.1670	0.0316	1.48E-07
**IL7**	0.0994	0.0262	1.52E-04
**IL8**	0.0880	0.0266	9.62E-04
**LAP.TGF.beta.1**	0.0968	0.0256	1.59E-04
**LIF.R**	-0.0571	0.0262	2.91E-02
**MCP.1**	0.0681	0.0260	8.95E-03
**MCP.2**	0.0770	0.0260	3.14E-03
**MCP.4**	0.0438	0.0257	8.89E-02
**MMP.1**	0.0772	0.0252	2.22E-03
**MMP.10**	0.0814	0.0256	1.50E-03
**NT.3**	-0.0534	0.0252	3.40E-02
**OPG**	0.0271	0.0271	3.18E-01
**OSM**	0.2153	0.0255	<2.00E-16
**PD.L1**	0.0894	0.0254	4.46E-04
**SCF**	-0.0109	0.0260	6.76E-01
**SIRT2**	0.0315	0.0280	2.61E-01
**SLAMF1**	0.0670	0.0266	1.18E-02
**ST1A1**	0.0743	0.0257	3.93E-03
**STAMBP**	0.0495	0.0258	5.51E-02
**TGF.alpha**	0.1283	0.0259	8.21E-07
**TNF**	0.1054	0.0255	3.75E-06
**TNFB**	-0.0168	0.0252	5.06E-01
**TNFRSF9**	0.0455	0.0263	8.36E-02
**TNFSF14**	0.2049	0.0251	7.07E-16
**TRAIL**	0.0230	0.0258	3.72E-01
**TRANCE**	0.0671	0.0255	8.68E-03
**TWEAK**	-0.1278	0.0258	7.81E-07
**uPA**	-0.0931	0.0262	3.94E-04
**VEGFA**	0.1965	0.0259	5.32E-14
**X4E.BP1**	0.0511	0.0253	4.39E-02

Correlations were adjusted for age and standardized.

Of the 70 correlations, 44 are significantly correlated with GlycA

Table S3. Correlations between hsCRP and inflammatory mediators

Variable	hsCRP correlations	hsCRP SE	hsCRP p-values
**ADA**	-0.0204	0.0286	4.77E-01
**AXIN1**	0.0520	0.0282	6.52E-02
**CASP.8**	0.0073	0.0282	7.95E-01
**CCL11**	-0.0022	0.0996	9.42E-01
**CCL19**	0.1462	0.0274	1.10E-07
**CCL20**	0.0856	0.0274	1.84E-03
**CCL23**	0.0516	0.0285	7.06E-02
**CCL25**	-0.0015	0.0300	9.61E-01
**CCL28**	-0.0280	0.0296	3.44E-01
**CCL3**	0.1343	0.0285	2.59E-06
**CCL4**	0.0997	0.0283	4.46E-04
**CD244**	0.0492	0.2821	8.16E-02
**CD40**	0.0828	0.0283	3.45E-03
**CD5**	0.0755	0.0281	7.29E-03
**CD6**	0.0147	0.0287	6.09E-01
**CD8A**	0.0563	0.0273	3.97E-02
**CDCP1**	0.1168	0.0330	4.20E-04
**CSF.1**	0.1470	0.0283	2.39E-07
**CST5**	0.0316	0.0293	2.82E-01
**CX3CL1**	-0.0419	0.0281	1.36E-01
**CXCL1**	0.0472	0.0282	9.38E-02
**CXCL10**	0.0502	0.0288	8.15E-02
**CXCL11**	0.0956	0.0276	5.54E-04
**CXCL5**	0.0405	0.0280	1.49E-01
**CXCL6**	0.0346	0.0282	2.21E-01
**CXCL9**	0.0579	0.0319	6.98E-02
**DNER**	-0.1426	0.0282	4.75E-07
**EN.RAGE**	0.0918	0.0285	1.29E-04
**FGF.19**	0.0286	0.0285	3.17E-01
**FGF.21**	0.0614	0.0283	3.02E-02
**Flt3L**	0.0069	0.0293	8.13E-01
**HGF**	0.1477	0.0297	7.31E-07
**IFN.gamma**	0.0551	0.0283	5.19E-02
**IL.10RB**	0.1036	0.0281	2.37E-04
**IL.12B**	0.0704	0.0283	1.29E-02

**IL.15RA**	0.0953	0.0322	3.08E-03
**IL.17C**	0.0682	0.0311	2.85E-02
**IL.18R1**	0.1618	0.0274	4.29E-09
**IL10**	0.1048	0.0287	2.68E-04
**IL18**	0.0594	0.0282	3.54E-02
**IL6**	0.2553	0.0382	3.81E-11
**IL7**	0.0563	0.0290	5.24E-02
**IL8**	0.0491	0.2960	9.72E-02
**LAP.TGF.beta.1**	0.0906	0.0282	1.33E-03
**LIF.R**	0.0022	0.0286	9.39E-01
**MCP.1**	0.0625	0.0291	3.17E-02
**MCP.2**	0.0319	0.0288	1.74E-01
**MCP.4**	0.0081	0.0285	7.78E-01
**MMP.1**	0.0660	0.0279	1.79E-02
**MMP.10**	0.0551	0.0282	5.05E-02
**NT.3**	-0.0219	0.0277	4.30E-01
**OPG**	0.0698	0.0301	2.38E-02
**OSM**	0.1539	0.0283	6.22E-08
**PD.L1**	0.0760	0.0279	6.60E-03
**SCF**	-0.0328	0.0282	2.45E-01
**SIRT2**	0.0163	0.0313	6.02E-01
**SLAMF1**	0.1030	0.0297	5.50E-04
**ST1A1**	0.0089	0.0287	7.57E-01
**STAMBP**	0.0295	0.0283	2.96E-01
**TGF.alpha**	0.0784	0.0285	6.00E-03
**TNF**	0.1137	0.0282	5.93E-05
**TNFB**	-0.0114	0.0278	6.82E-01
**TNFRSF9**	0.1023	0.0290	4.42E-04
**TNFSF14**	0.1305	0.0280	3.51E-06
**TRAIL**	0.0742	0.0285	9.22E-03
**TRANCE**	0.0097	0.0281	7.29E-01
**TWEAK**	-0.1172	0.0285	3.97E-05
**uPA**	-0.0063	0.0290	8.29E-01
**VEGFA**	0.1587	0.0289	4.65E-08
**X4E.BP1**	0.0236	0.0280	4.00E-01

Correlations were adjusted for age and standardized.

Of the 70 correlations, 36 are significantly correlated with hsCRP

Table S4. PRS results for GlycA and CRP

Base. GWAS	Threshold	PRS.R2	Full.R2	Null.R2	Coefficient	Stand Error	P	Num_SNP
**GlycA – target phenotype**								
**B_NGF**	0.3904	0.0047	0.0167	0.0120	-197.16	69.70	4.73E-03	86767
**CRP**	0.0525	0.0084	0.0205	0.0120	479.34	125.94	1.46E-04	24677
**CTACK**	0.0004	0.0015	0.0136	0.0120	-3.80	2.35	1.06E-01	321
**EOTAXIN**	0.0076	0.0015	0.0136	0.0120	-25.62	15.75	1.04E-01	4562
**FGF_BASIC**	0.0005	0.0018	0.0138	0.0120	6.86	3.95	8.25E-02	390
**GCSF**	0.2646	0.0029	0.0150	0.0120	-191.20	85.64	2.57E-02	69225
**GROA**	0.0026	0.0045	0.0166	0.0120	16.04	5.76	5.43E-03	1632
**HGF**	0.0047	0.0032	0.0152	0.0120	29.60	12.68	1.97E-02	3025
**IFN_gamma**	0.0301	0.0008	0.0128	0.0120	34.43	29.48	2.43E-01	13752
**IL10**	0.3433	0.0030	0.0151	0.0120	-212.77	93.28	2.27E-02	81302
**IL12**	0.1460	0.0049	0.0170	0.0120	-184.79	63.64	3.74E-03	46700
**IL13**	0.0009	0.0012	0.0133	0.0120	-5.42	3.73	1.46E-01	721
**IL16**	0.0003	0.0032	0.0153	0.0120	3.93	1.67	1.86E-02	186
**IL17**	0.0003	0.0032	0.0152	0.0120	-7.56	3.23	1.95E-02	277
**IL18**	0.0000	0.0023	0.0144	0.0120	0.23	0.11	4.64E-02	6
**IL1B**	0.0069	0.0025	0.0145	0.0120	-19.80	9.65	4.03E-02	3979
**IL1RA**	0.0001	0.0014	0.0134	0.0120	1.22	0.80	1.25E-01	41
**IL2**	0.0001	0.0019	0.0140	0.0120	2.20	1.21	7.00E-02	93
**IL2RA**	0.0021	0.0019	0.0139	0.0120	9.66	5.41	7.43E-02	1384
**IL4**	0.0174	0.0027	0.0147	0.0120	-49.91	23.22	3.17E-02	8899
**IL5**	0.0002	0.0050	0.0171	0.0120	4.56	1.55	3.38E-03	144
**IL6**	1.0000	0.0017	0.0138	0.0120	-256.67	149.02	8.52E-02	133811
**IL7**	0.0679	0.0029	0.0150	0.0120	-65.97	29.43	2.51E-02	25883
**IL8**	0.0017	0.0020	0.0140	0.0120	-9.39	5.12	6.67E-02	1209
**IL9**	0.0504	0.0023	0.0143	0.0120	-53.39	27.05	4.85E-02	20691
**IP10**	0.0005	0.0013	0.0134	0.0120	-3.87	2.55	1.30E-01	386
**MCP1**	0.0833	0.0010	0.0131	0.0120	-64.51	48.49	1.84E-01	30681
**MCP3**	0.0035	0.0052	0.0173	0.0120	-11.94	3.98	2.74E-03	2199
**MCSF**	0.0001	0.0038	0.0158	0.0120	-1.93	0.76	1.08E-02	59
**MIF**	0.0001	0.0008	0.0129	0.0120	1.11	0.94	2.35E-01	60
**MIG**	0.0487	0.0048	0.0168	0.0120	-72.30	25.28	4.29E-03	20112
**MIP1A**	0.0003	0.0043	0.0164	0.0120	5.47	2.00	6.44E-03	248

**MIP1B**	0.2459	0.0012	0.0133	0.0120	-115.26	79.88	1.49E-01	65930
**PDGF**	0.0449	0.0029	0.0150	0.0120	86.18	38.51	2.53E-02	19340
**RANTES**	0.0013	0.0021	0.0141	0.0120	-8.34	4.42	5.96E-02	959
**SCF**	0.0001	0.0013	0.0134	0.0120	-2.00	1.32	1.31E-01	50
**SCGF**	0.0004	0.0042	0.0162	0.0120	6.80	2.54	7.43E-03	350
**SDF1A**	0.0953	0.0016	0.0137	0.0120	-83.10	50.02	9.68E-02	33579
**TNF_A**	0.0032	0.0042	0.0162	0.0120	-17.93	6.68	7.36E-03	2036
**TNF_B**	0.0051	0.0023	0.0143	0.0120	-11.39	5.78	4.88E-02	2834
**TRAIL**	0.0700	0.0019	0.0140	0.0120	85.59	47.24	7.02E-02	27026
**VEGF**	0.0049	0.0025	0.0146	0.0120	-23.60	11.32	3.71E-02	3167
**CRP – target phenotype**								
**B_NGF**	0.0001	0.0005	0.0013	0.0008	38.01	25.75	1.40E-01	84
**CRP**	0.0001	0.0071	0.0079	0.0008	864.20	155.34	2.81E-08	507
**CTACK**	0.0122	0.0013	0.0021	0.0008	737.42	315.68	1.95E-02	6432
**EOTAXIN**	0.0025	0.0007	0.0015	0.0008	361.04	208.77	8.38E-02	1784
**FGF_BASIC**	0.0044	0.0004	0.0012	0.0008	-358.94	277.27	1.96E-01	2841
**GCSF**	0.0002	0.0013	0.0022	0.0008	151.87	63.17	1.62E-02	182
**GROA**	0.0001	0.0017	0.0026	0.0008	32.43	11.76	5.83E-03	42
**HGF**	0.0388	0.0022	0.0030	0.0008	2526.22	819.79	2.07E-03	16920
**IFN_gamma**	0.0001	0.0004	0.0012	0.0008	-34.84	27.18	2.00E-01	43
**IL10**	0.0063	0.0014	0.0022	0.0008	816.14	331.20	1.38E-02	3851
**IL12**	0.0000	0.0008	0.0016	0.0008	11.29	6.21	6.89E-02	13
**IL13**	0.0098	0.0018	0.0026	0.0008	793.16	286.06	5.58E-03	5469
**IL16**	0.1295	0.0003	0.0011	0.0008	1075.56	944.12	2.55E-01	41281
**IL17**	0.0002	0.0004	0.0012	0.0008	79.30	63.41	2.11E-01	192
**IL18**	0.1069	0.0004	0.0013	0.0008	-1213.68	900.54	1.78E-01	36606
**IL1B**	0.0009	0.0006	0.0014	0.0008	121.57	74.53	1.03E-01	608
**IL1RA**	0.0020	0.0021	0.0029	0.0008	390.15	128.89	2.48E-03	1435
**IL2**	0.0015	0.0007	0.0015	0.0008	-187.34	106.85	7.96E-02	1027
**IL2RA**	0.3143	0.0006	0.0014	0.0008	-2375.38	1487.98	1.10E-01	76236
**IL4**	0.0126	0.0008	0.0016	0.0008	-864.17	473.95	6.83E-02	6744
**IL5**	0.0000	0.0011	0.0019	0.0008	4.33	2.01	3.12E-02	1
**IL6**	0.0003	0.0012	0.0020	0.0008	-151.93	67.34	2.41E-02	209
**IL7**	0.0023	0.0013	0.0021	0.0008	311.91	132.34	1.85E-02	1592
**IL8**	0.0281	0.0002	0.0011	0.0008	-480.02	466.23	3.03E-01	12903
**IL9**	0.0192	0.0009	0.0017	0.0008	773.62	392.08	4.85E-02	9498
**IP10**	0.0157	0.0007	0.0015	0.0008	-627.41	357.22	7.91E-02	8275
**MCP1**	0.0954	0.0011	0.0019	0.0008	-2693.09	1229.10	2.85E-02	33867
**MCP3**	0.0001	0.0006	0.0014	0.0008	-24.82	15.29	1.05E-01	96
**MCSF**	0.0784	0.0008	0.0016	0.0008	-1195.01	652.87	6.73E-02	28887

**MIF**	0.0044	0.0002	0.0011	0.0008	196.45	191.37	3.05E-01	2798
**MIG**	0.0000	0.0011	0.0020	0.0008	6.92	3.13	2.71E-02	2
**MIP1A**	0.0154	0.0010	0.0019	0.0008	727.29	345.39	3.53E-02	7966
**MIP1B**	0.0005	0.0006	0.0014	0.0008	-107.63	68.69	1.17E-01	585
**PDGF**	0.0148	0.0006	0.0015	0.0008	-868.73	531.28	1.02E-01	8100
**RANTES**	0.3018	0.0005	0.0014	0.0008	2172.89	1422.49	1.27E-01	74301
**SCF**	0.0007	0.0008	0.0017	0.0008	-219.66	116.39	5.92E-02	540
**SCGF**	0.0001	0.0008	0.0017	0.0008	-42.38	22.26	5.71E-02	65
**SDF1A**	0.0358	0.0008	0.0017	0.0008	-1469.06	764.70	5.48E-02	15848
**TNF_A**	0.0045	0.0013	0.0022	0.0008	-449.89	186.96	1.62E-02	2691
**TNF_B**	0.0019	0.0010	0.0018	0.0008	-162.52	79.27	4.04E-02	1146
**TRAIL**	0.0011	0.0018	0.0026	0.0008	-391.97	141.91	5.77E-03	857
**VEGF**	0.0082	0.0006	0.0014	0.0008	-547.90	348.68	1.16E-01	4841

Table S5. MR GlycA as exposure using the IVW method

**Outcome**	**Instrumental Variables**	**Estimate**	**SE**	**95% Confidence Interval**	**P-value**	**Heterogeneity**
**hsCRP**	21	0.085	0.024	0.039, 0.132	<0.001	0.1698
**FGF**	38	0.276	0.063	0.153, 0.399	<0.001	0.6936
**HGF**	41	0.375	0.064	0.250, 0.500	<0.001	0.6368
**IL6**	41	0.266	0.060	0.147, 0.384	<0.001	0.7227
**IL7**	36	0.380	0.095	0.195, 0.566	<0.001	0.6706
**TNFA**	37	0.301	0.088	0.129, 0.474	0.001	0.9659
**VEGF**	45	0.377	0.074	0.232, 0.523	<0.001	0.9801

Table S6. MR hsCRP as the exposure, using the IVW method

Outcome	Instrumental Variables	Estimate	SE	95% Confidence Interval	P-value	Heterogeneity
**HGF**	34	1.222	0.102	1.022, 1.422	<0.001	1.0000
**IL6**	46	0.276	0.052	0.174, 0.379	<0.001	0.8245
**IL10**	38	0.360	0.072	0.218, 0.502	<0.001	0.9966
**TNFA**	35	0.554	0.119	0.321, 0.787	<0.001	0.9999
**VEGF**	35	0.274	0.067	0.143, 0.405	<0.001	0.9442

Table S7. Gene Ontology results: enrichment analysis r for GlycA and CRP variables for gene ontology (GO) terms

GO ID (Term)	Class	Variable	Rank (Node)	Annotated	Significant	Expected	p-value (Fisher test)
**GO:0007156 (Homophilic cell adhesion via plasma membrane)**	BP	GlycA	1	163	147	123.6	3.60E-06
		CRP	1	160	133	110.7	1.40E-05
**GO:0005515 (protein binding)**	MF	GlycA	1	13852	10967	10836.8	4.00E-08
		CRP	2	13581	9564	9406.4	5.90E-07
**GO:0005829 (cytosol)**	CC	GlycA	1	5137	4156	3898.3	1.00E-25
		CRP	1	4984	3673	3443.7	2.50E-19

GO terms are found in three classes: biological process (BP), molecular function (MF), and cellular component (CC).
